# Case Report: Ultra-early tangential excision combined with negative pressure wound therapy as a novel surgical detoxification strategy for severe hydrofluoric acid burns complicated by intractable hypocalcemia

**DOI:** 10.3389/ftox.2026.1859979

**Published:** 2026-06-03

**Authors:** Xiaokun Tian, Bin Xu, Kai Zhang, Chen Sun, Cheng Wang, Haiyan Shi

**Affiliations:** 1 Department of Burn, Plastic and Wound Repair, The First Hospital of Zibo, Zibo, Shandong, China; 2 Department of Blood Transfusion, The First Hospital of Zibo, Zibo, Shandong, China

**Keywords:** early tangential excision, hydrofluoric acid burn, hypocalcemia, negative pressure wound therapy, urinary fluoride

## Abstract

**Introduction:**

Severe hydrofluoric acid (HF) burns are life-threatening due to the rapid systemic absorption of fluoride ions, which precipitate profound hypocalcemia and fatal arrhythmias. Traditional conservative neutralization therapies often fail to halt the continuous absorption of toxins from deep necrotic tissues in high-concentration exposures.

**Case Presentation:**

We report a 40-year-old male presenting with severe HF burns (20% total body surface area) following exposure to 40% HF. The patient rapidly developed intractable hypocalcemia (Ca^2+^ 0.48 mmol/L) and experienced 12 episodes of ventricular fibrillation within 8 h. Alongside intensive medical resuscitation, an emergency tangential excision of the burn eschar was performed merely 2 h post-injury to achieve immediate “source control”, synergistically coupled with Negative Pressure Wound Therapy (NPWT). Dynamic urinary fluoride monitoring confirmed a rapid decline in the systemic toxic load following the surgical intervention. The patient survived the critical phase, underwent staged skin grafting, and was discharged with complete clinical recovery and fully preserved physical mobility at the 1-year follow-up.

**Conclusion:**

Proactive surgical source control through extremely early tangential excision, combined with NPWT, serves as a novel surgical detoxification strategy that effectively severs the continuous systemic absorption of fluoride. This strategy, validated by dynamic urinary fluoride monitoring, significantly improves survival in severe HF burns and warrants broader clinical adoption.

## 1 Introduction

Hydrofluoric acid (HF) is a highly corrosive inorganic acid widely utilized in industrial manufacturing. Unlike typical thermal or chemical burns, HF exposure presents a dual and severe threat: the hydrogen ions cause profound local tissue liquefaction and necrosis, while the highly penetrative fluoride ions are rapidly absorbed into the systemic circulation (Lippert et al., 2020; Wan et al., 2019). Once absorbed, fluoride ions bind precipitously with endogenous calcium and magnesium, precipitating intractable hypocalcemia, hypomagnesemia, and metabolic acidosis. This severe electrolyte derangement can swiftly culminate in malignant cardiac arrhythmias, representing the primary cause of early mortality in affected patients (Yu et al., 2020; Bajraktarova-Valjakova et al., 2018).

The conventional management paradigm for HF burns primarily centers on copious water irrigation followed by the topical and systemic administration of calcium to neutralize the absorbed fluoride. However, in cases of severe burns involving high concentrations of HF, these conservative neutralization strategies are often therapeutically inadequate. Intravenous calcium replacement merely acts as a passive countermeasure in the bloodstream and fails to arrest the continuous release and systemic absorption of fluoride ions sequestered within the deep necrotic tissues of the wound bed. Consequently, despite aggressive medical resuscitation, patients remain at imminent risk of fatal systemic fluorosis.

To address this critical therapeutic gap, proactive intervention targeting the “toxin source” is imperative. Early tangential excision of the burn eschar aims to physically eradicate the fluoride-laden necrotic tissue, thereby definitively halting the continuous absorption of the toxin. Furthermore, coupling this surgical approach with Negative Pressure Wound Therapy (NPWT) can synergistically enhance the continuous clearance of fluoride-rich exudate and optimize the local wound microenvironment. Herein, we report the successful management of a 40-year-old male presenting with severe HF burns (20% total body surface area) and life-threatening hypocalcemia. By implementing early escharectomy combined with NPWT, systemic toxicity was effectively controlled. This case highlights the crucial role of immediate surgical source control and dynamic monitoring in averting mortality following severe HF exposure.

## 2 Case description

### 2.1 Patient information and clinical findings

A 40-year-old previously healthy male factory worker presented to the emergency department 40 min after accidental exposure to 40% hydrofluoric acid (HF) at work. Immediate first aid at the scene involved cold water irrigation for approximately 20 min. Upon admission, the patient was unconscious, profoundly restless, and hemodynamically unstable with a blood pressure of 99/56 mmHg, a heart rate of 121 beats/min, and an oxygen saturation of 85%. Physical examination revealed burn wounds covering 20% of his total body surface area (TBSA), predominantly distributed across the head, face, trunk, and extremities. The burns were categorized as deep partial-thickness to full-thickness (deep II-III degree), with the majority of the wound bed presenting a dark gray, leathery appearance with complete loss of sensation and no bleeding upon pinprick.

### 2.2 Diagnostic assessment

Initial arterial blood gas and laboratory analyses demonstrated severe systemic toxicity and metabolic derangements. Key findings included profound hypocalcemia (Ca^2+^ 0.48 mmol/L) and hypomagnesemia (Mg^2+^ 0.55 mmol/L), accompanied by severe metabolic acidosis (pH 7.19, HCO_3_^−^ 14.9 mmol/L) and hyperlactatemia (Lactate 9.3 mmol/L).

### 2.3 Therapeutic interventions

**FIGURE 1:**
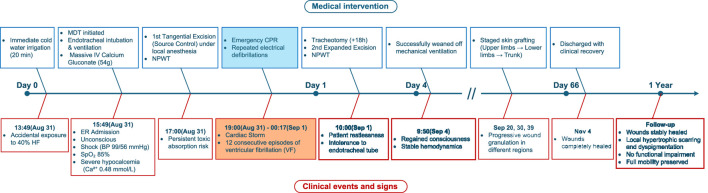
Clinical timeline of the multidisciplinary rescue and surgical interventions for the severe hydrofluoric acid burn patient. The timeline details the progression of clinical events (below the axis) and corresponding therapeutic interventions (above the axis) from the time of injury to hospital discharge. Notably, a “cardiac storm” comprising 12 episodes of ventricular fibrillation occurred within a 5-h window post-admission, successfully managed with repeated defibrillation. Proactive “source control” was achieved through two crucial stages of early tangential excision combined with Negative Pressure Wound Therapy (NPWT). (Abbreviations: HF, hydrofluoric acid; ER, emergency room; MDT, multidisciplinary team; VF, ventricular fibrillation; NPWT, negative pressure wound therapy; CPR, cardiopulmonary resuscitation).

Immediate Resuscitation (Admission): A multidisciplinary team (MDT) was immediately mobilized (Figure 1). The patient underwent prompt endotracheal intubation, mechanical ventilation, and sedation. Aggressive intravenous supplementation of calcium gluconate (totaling 54 g on the first day) was immediately initiated, alongside topical calcium gluconate infiltration into the wounds to passively neutralize the absorbed fluoride. Given the extreme urgency of the profound hypocalcemia and recurrent ventricular fibrillation, the ultra-early resuscitation strictly prioritized rapid and massive calcium replacement to stabilize the fatal arrhythmias. Magnesium and potassium supplementation were subsequently administered during the intensive care stabilization phase after the initial cardiac storms were controlled.

Surgical Source Control (+2 h): To proactively halt the continuous systemic absorption of fluoride ions, an emergency bedside tangential excision of the full-thickness eschar on the left shoulder and left lower extremity was performed just 2 h post-injury. Given the patient’s severe hemodynamic instability and recurrent ventricular fibrillation, which precluded general anesthesia and transport to the operating room, this procedure was conducted at the bedside under local anesthesia. This was immediately followed by the application of Negative Pressure Wound Therapy (NPWT).

Cardiac Complications (+2 to +10 h): Despite aggressive systemic neutralization and initial surgical debridement, the sheer magnitude of the initial fluoride load precipitated 12 episodes of ventricular fibrillation within the first 8 h post-operation, all of which were successfully managed with emergency electrical defibrillation.

Secondary Excision (+18 h): Due to persistent restlessness and intolerance to the endotracheal tube at 18 h post-admission, a tracheotomy was performed. Concurrently, a secondary expanded excision of deeper necrotic tissues was conducted under local anesthesia, again coupled with continuous NPWT.

### 2.4 Follow-up and outcomes

**FIGURE 2:**
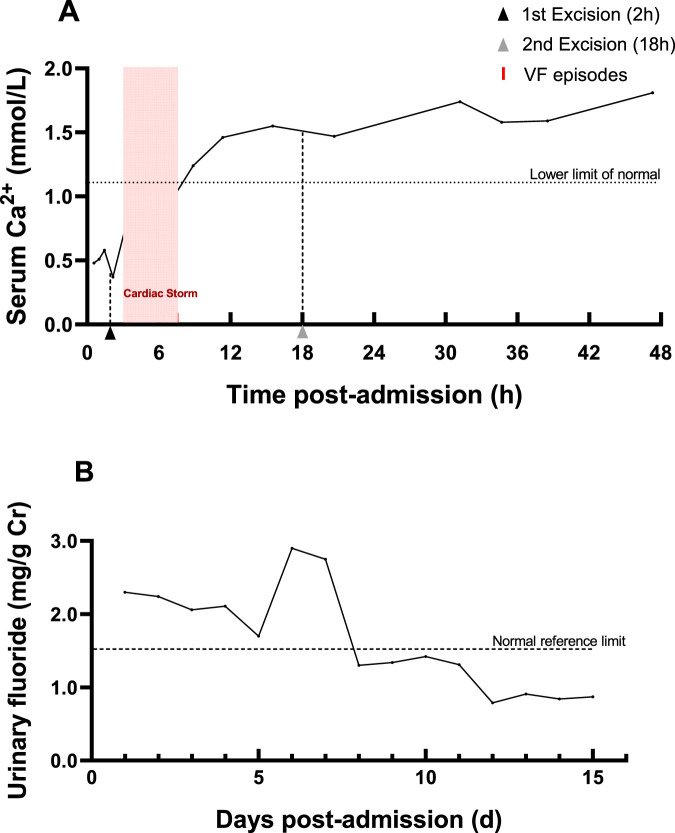
Dynamic changes in serum ionized calcium and urinary fluoride concentrations. **(A)** Serum ionized calcium levels during the first 48 h. The horizontal dashed line represents the lower limit of the normal reference range (1.15 mmol/L). Red markers indicate the 12 episodes of ventricular fibrillation (VF) that occurred during the profound hypocalcemic phase. A rapid recovery of calcium levels followed by the cessation of cardiac events was observed after the first tangential excision. **(B)** Urinary fluoride concentrations during the first 15 days post-admission. The dashed line represents the normal reference limit. A significant and continuous decline in systemic fluoride burden was achieved, which paralleled the stabilization of the patient’s clinical condition following early surgical interventions. (Abbreviations: Ca^2+^, ionized calcium).

**FIGURE 3:**
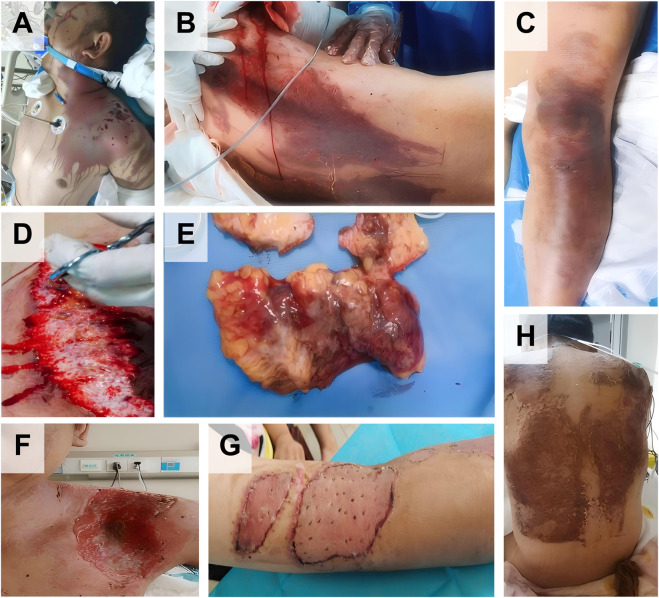
Clinical progression and surgical management of severe hydrofluoric acid burns. **(A–C)** Clinical presentation at admission, showing extensive deep partial-thickness to full-thickness burns with characteristic dark gray, leathery eschars on the head, neck, and anterior trunk **(A)**, the posterior trunk **(B)**, and the lower extremity **(C)**. **(D)** The viable wound bed immediately following early tangential excision. **(E)** The excised necrotic eschar, effectively removing the tissue reservoir of unabsorbed fluoride to achieve proactive “source control.” **(F)** Healthy granulation tissue formation on the shoulder wound following Negative Pressure Wound Therapy (NPWT), providing an optimal microenvironment for subsequent grafting. **(G)** Well-taken split-thickness skin grafts on the upper extremity. **(H)** Completely epithelialized and stably healed wounds on the posterior trunk.

Following the surgical interventions, strict dynamic monitoring of serum calcium and urinary fluoride concentrations was maintained, demonstrating a rapid decline in toxic load correlating with clinical stabilization (Figure 2). The patient regained consciousness and was successfully weaned off mechanical ventilation on day 6 post-admission. Under continuous NPWT, infection control, and systemic nutritional support, the wound bed conditions steadily improved. Three staged skin grafting procedures were successfully executed on days 20, 30, and 39 post-injury to achieve complete wound closure (Figure 3). The patient was ultimately discharged after 66 days of hospitalization with complete clinical recovery. At the 1-year outpatient follow-up, the patient maintained an excellent recovery status with all wounds stably healed. Although local hypertrophic scarring and dyspigmentation were observed at the affected sites, the patient exhibited no joint contractures or functional impairments, and his physical mobility was fully preserved. From the patient’s perspective, he expressed profound gratitude for the rapid intervention, noting that while the initial pain was excruciating, the subsequent skin grafting and rehabilitation allowed him to return to a normal life and independently perform daily activities, significantly exceeding his initial expectations. The hypertrophic scarring was attributed to the prolonged deep tissue inflammation characteristic of chemical burns. During the 1-year rehabilitation phase, continuous pressure garment therapy and topical silicone gel applications were implemented to mitigate scar contracture and preserve joint mobility.

## 3 Discussion

Hydrofluoric acid (HF) is a highly corrosive agent that causes severe tissue liquefaction and unique systemic toxicity due to the rapid absorption of fluoride ions. The severity of the injury is directly proportional to the HF concentration (Mao et al., 2022). In this case, exposure to 40% HF precipitated intractable hypocalcemia as fluoride ions rapidly chelated endogenous calcium and magnesium. The subsequent occurrence of 12 episodes of ventricular fibrillation within a 5-h window starkly illustrates the lethal myocardial electrophysiological derangements triggered by this massive toxic load.

While early multidisciplinary life support—including massive intravenous calcium supplementation and mechanical ventilation—stabilized the patient, conventional systemic neutralization alone is often insufficient (Hu et al., 2024; Haruta and Mandell, 2023). Intravenous calcium merely neutralizes circulating fluoride but fails to halt its continuous release from deep necrotic tissues. Therefore, the clinical breakthrough in this case was the proactive implementation of surgical “source control”. By performing an emergency tangential excision just 2 h post-injury, we physically eradicated the fluoride-rich eschar, thereby definitively severing the route of continuous toxic absorption (Zhang et al., 2022; Han et al., 2017). Furthermore, the concurrent application of Negative Pressure Wound Therapy (NPWT) exerted a powerful synergistic therapeutic effect. NPWT not only continuously drained the fluoride-laden exudate to prevent local accumulation but also improved microvascular circulation in the wound bed, enhancing the efficacy of topically applied calcium and preparing the site for subsequent skin grafting (Kement and Başkıran, 2018).

The efficacy of this early surgical intervention was objectively validated by dynamic urinary fluoride monitoring. The rapid decline in urinary fluoride concentration, which closely paralleled the restoration of serum calcium levels, confirms the decisive role of escharectomy in reducing the total systemic fluoride burden (Idowu et al., 2019). Notably, while Continuous Renal Replacement Therapy (CRRT) is highly effective at clearing free circulating fluoride (Bai and Gao, 2025), our patient survived without it. This highlights a crucial clinical distinction: CRRT predominantly clears blood-borne toxins, whereas early surgical excision directly addresses tissue-bound fluoride at a higher efficiency (Ricketts and Kimble, 2003). In resource-limited settings or for patients where CRRT is contraindicated, early excision combined with NPWT presents a universally applicable and highly effective strategy. For patients with ultra-large area burns or acute kidney injury, the combination of both modalities may further improve survival rates.

Looking forward, to further mitigate the catastrophic consequences of HF exposure, significant efforts must be directed toward translational research and the upgrading of detoxification and protective technologies. There is an urgent clinical demand for the development of highly effective, domestically produced antidotes, such as cost-effective Hexafluorine analogs, novel calcium/magnesium chelating hydrogels or advanced wound dressings, and targeted injectable nano-antidotes. Furthermore, the advancement of smart personal protective equipment (PPE) and environmental monitoring is critical. Innovations including wearable fluoride ion (F^−^) exposure sensors, automated emergency decontamination systems, and real-time chemical leakage warning networks will substantially reduce occupational hazards.

More importantly, given the extreme toxicity and narrow therapeutic window of HF burns, establishing a specialized, tiered regional rescue network is paramount. Under the unified deployment of municipal health commissions, a comprehensive continuum of care must be constructed, integrating point-of-injury first aid (industrial sites), grassroots healthcare facilities, county hospitals, and specialized burn centers. Establishing dedicated HF burn treatment centers in high-risk industrial zones, alongside dispatching emergency burn experts to conduct specialized training for frontline grassroots physicians, will ensure rapid triage, early correct intervention, and optimal definitive care, thereby fundamentally improving the regional survival rates of such devastating injuries.

## 4 Conclusion

The successful management of severe HF burns complicated by life-threatening hypocalcemia necessitates a paradigm of “rapid assessment, multidisciplinary collaboration, proactive source control, and comprehensive support”. Early tangential excision combined with NPWT effectively halts systemic fluoride absorption, while dynamic urinary fluoride monitoring serves as a crucial, objective prognostic tool. This proactive surgical detoxification strategy significantly reduces mortality and merits broader clinical application. Ultimately, beyond definitive clinical management, mitigating the devastating impact of HF exposure relies on broader systemic efforts: the ongoing translational development of novel targeted antidotes, the implementation of smart industrial monitoring technologies, and the establishment of a robust, tiered regional rescue network to ensure a seamless continuum of care from the frontline to specialized centers.

## Data Availability

The original contributions presented in the study are included in the article/supplementary material, further inquiries can be directed to the corresponding authors.
